# Balancing Surgery and Radiosurgery in Jugulotympanic Paragangliomas

**DOI:** 10.7759/cureus.87609

**Published:** 2025-07-09

**Authors:** Giorgos Sideris, Evangelos Panagoulis, Ilias Lazarou, Nikolaos Papadimitriou, Alexander Delides, Dimitrios Palantzas, Panagiotis P Gogoulos, George Korres, Petros V Vlastarakos, Thomas Nikolopoulos

**Affiliations:** 1 2nd ENT Department, Attikon University Hospital, National and Kapodistrian University of Athens, Athens, GRC; 2 2nd ENT Department, Attikon University Hospital, School of Medicine, National and Kapodistrian University of Athens, Athens, GRC; 3 2nd Otolaryngology Department, Attikon University Hospital, School of Medicine, National and Kapodistrian University of Athens, Athens, GRC

**Keywords:** glomus jugulare, glomus tumor, glomus tympanicum, stereotactic radiosurgery, surgical excision

## Abstract

Jugulotympanic paragangliomas (JTPs) are rare, benign, but locally aggressive neuroendocrine tumors of the temporal bone and jugular foramen. Due to their proximity to critical neurovascular structures, they can cause considerable morbidity. This study retrospectively assesses treatment outcomes in JTP patients, examining the effectiveness of surgical resection and stereotactic radiosurgery (RS) in tumor control and recurrence prevention. A retrospective analysis was performed on 11 adult patients diagnosed with JTP from January 2022 to December 2024. Data collected included demographics, tumor characteristics, surgical approach, histopathology, and follow-up imaging. Tumors were classified using the Fisch system, and the Ki-67 index was used to assess proliferative activity. Eleven patients (seven females and four males; median age = 64.5 years) were included. Tumor sizes ranged from 1.0 × 0.5 cm to 2.7 × 2.2 cm. Based on the Fisch classification, six tumors were class B, two were class A, one was class C1, and one was class D1. All patients underwent surgical excision via an endoaural-transcanal approach with potassium titanyl phosphate (KTP) laser hemostasis. Complete resection was achieved in five cases; six required additional stereotactic RS. Follow-up imaging showed no recurrence in fully resected cases, and tumor control in those treated with RS. Most tumors had a Ki-67 proliferation index < 5%. Surgical resection remains the preferred treatment for small, accessible JTPs. For most glomus jugulare cases, as well as residual or surgically complex tumors, a combination of surgery and RS plays a crucial role in effective management. The Ki-67 proliferation index does not independently predict recurrence.

## Introduction

Glomus tumors, also known as paragangliomas, are rare, highly vascular neuroendocrine tumors with an estimated incidence of one case per million persons, commonly presenting in the 5th decade of life, that primarily arise in the temporal bone, carotid body, or jugular bulb [1,2]. While typically benign, their slow-growing nature and proximity to critical neurovascular structures can lead to significant morbidity.

The primary subtypes of glomus tumors in the head and neck region are glomus tympanicum and glomus jugulare. Glomus tympanicum, originating from paraganglionic cells in the middle ear, is the most common benign tumor of this region. Patients typically present with pulsatile tinnitus and conductive hearing loss due to the tumor’s proximity to the tympanic membrane and ossicles [3-5]. Glomus jugulare tumors, arising at the jugular foramen, tend to be larger and more invasive than glomus tympanicum. They can extend to involve cranial nerves and the skull base, leading to symptoms such as lower cranial nerve palsies, hoarseness, dysphagia, and sometimes conductive hearing loss [6,7]. The preferred pathological term for both glomus jugulare and glomus tympanicum is jugulotympanic paragangliomas (JTPs), reflecting the similar underlying pathophysiology behind them.

The management strategy for JTP depends on tumor size, location, growth rate, and patient health status. Treatment options range from observation (for asymptomatic or slow-growing cases) to surgical resection, conventional fractionated radiotherapy (RT), stereotactic radiosurgery (RS) such as Gamma Knife, CyberKnife, and linear accelerator (LINAC)-based techniques, or a combined approach.

Traditionally, surgical resection has been the primary treatment for JTP. However, their vascularity and proximity to critical structures make complete excision challenging, especially for glomus jugulare tumors. In such cases, RS has emerged as a viable non-invasive alternative.

This study aims to analyze the clinical characteristics, treatment strategies, and outcomes of patients diagnosed with JTP in the middle ear and jugular foramen. By reviewing patient demographics, tumor location, surgical approaches, and follow-up imaging findings, the study evaluates the effectiveness of the classic treatment modalities, including surgery and RS. Additionally, it assesses tumor recurrence rates and the role of histological markers, such as the Ki-67 proliferation index, in predicting tumor behavior.

## Materials and methods

This retrospective observational study was conducted at Attikon University Hospital and was approved by the Institutional Review Board (Office for Human Research Protections Database Number: IORG0004614), with approval number IRB 404 (May 26, 2025). All patients included in the study provided written informed consent, and the analysis was conducted in accordance with the principles of the Declaration of Helsinki. The study involved the analysis of medical records, imaging, operative notes, and histopathological data from 11 adult patients with histologically confirmed JTP treated between January 2022 and December 2024.

Patient selection and data collection

Inclusion criteria encompassed patients aged ≥18 years with a confirmed diagnosis of glomus tympanicum or glomus jugulare tumor, based on clinical, radiological, and histopathological findings. Patients with incomplete records or those who had prior treatment for JTP at other institutions were excluded.

Clinical data collected included age, sex, presenting symptoms (e.g., pulsatile tinnitus, hearing loss, cranial nerve deficits), and comorbidities. Radiological assessments were used to determine tumor size, location, and involvement of surrounding structures.

Imaging and classification

Preoperative and follow-up imaging consisted of high-resolution computed tomography (HRCT) of the temporal bone and gadolinium-enhanced magnetic resonance imaging (MRI). HRCT scans were used to assess bony erosion, ossicular involvement, and jugular foramen widening. MRI, including T1, T2, and contrast-enhanced sequences, was used to evaluate soft tissue extent, vascularity, and skull base or intracranial extension.

Tumors were staged using the Fisch classification system, which categorizes lesions based on anatomic involvement and provides a framework for determining surgical complexity and planning [8].

Surgical procedure

All patients underwent surgical resection via a tailored approach based on tumor extent. The predominant technique used was the endoaural-transcanal approach under an operating microscope. In selected cases with more extensive lesions, a retroauricular extension or canal wall down approach was employed. A potassium titanyl phosphate (KTP) laser (532 nm wavelength) was used for precise intraoperative hemostasis, enabling effective coagulation of tumor-feeding vessels while maintaining a clear surgical field.

Ossicles infiltrated by the tumor were removed, most commonly the incus. Reconstruction using tympanoplasty techniques (type II or III, depending on the extent of ossicular chain disruption) was performed as needed. Care was taken to preserve the stapes and facial nerve whenever feasible. Facial nerve monitoring was employed in all cases.

Histopathological analysis

Tumor specimens were fixed in 10% formalin and processed using standard histological protocols. Hematoxylin and eosin staining confirmed the diagnosis of paraganglioma. Immunohistochemistry for synaptophysin and chromogranin A was used in selected cases to confirm neuroendocrine origin. The Ki-67 proliferation index was calculated by counting the percentage of positively stained tumor cell nuclei in high-power fields, serving as a marker of mitotic activity and potential biological aggressiveness.

Radiosurgery and follow-up

Patients with residual tumor postoperatively or with tumors involving critical structures were referred for RS. Treatment was delivered using either Gamma Knife or LINAC-based platforms, based on availability and anatomical suitability. RS planning was individualized, with dosing typically ranging between 12 and 18 Gy to the tumor margin in a single session or in three to five fractions, depending on tumor size and location.

Patients were followed up clinically and radiologically at three-month intervals during the first year and every six months thereafter. Follow-up imaging with HRCT and MRI was used to monitor for tumor recurrence or progression. Radiological response was categorized as stable disease, regression, or recurrence.

Statistical analysis

Due to the small sample size, descriptive statistics were used to summarize patient demographics, tumor characteristics, and surgical and radiological outcomes. Continuous variables were expressed as medians and ranges, and categorical variables as frequencies and percentages. No inferential statistical tests were performed, given the exploratory nature of this study.

## Results

The patients ranged in age from 46 to 76 years, with a median age of 64.5 years. Seven were female, and four were male. Tumor sizes varied, with the smallest measuring 1.0 x 0.5 cm and the largest measuring 2.7 x 2.2 cm. While some tumors remained confined to the tympanic cavity, epitympanum, and hypotympanum, as seen in case 5, without significant extension into adjacent structures, others exhibited involvement of the ossicles, facial recess, or minor erosion, as observed in cases 2 and 6. In more severe cases, tumors extended beyond the middle ear, infiltrating the jugular foramen, cochlear promontory, or even reaching intracranial structures. A notable example is case 7, where extensive invasion was observed. According to the Fisch classification, seven cases were categorized as B, while two fell into category A, one into category C1, and one into the D1 category (Table 1).

**Table 1:** Demographics and imaging findings.

Case	Age (years)	Sex	Tumor characteristics	Size	Fisch classification
1	54	M	Extending to the hypotympanum and epitympanum, with invasion of the eustachian tube and erosion of the stapes and tympanic structures.	1.2 x 0.6 cm (axial); 0.6 cm (cranial-caudal)	B
2	46	M	Occupying the hypotympanum, mesotympanum, and epitympanum. Involved ossicles and facial recess, minimal erosion of adjacent structures.	1.2 x 0.6 cm (axial); 1.6 cm (cranial-caudal)	B
3	56	F	Involving the epitympanum and tympanic cavity, causing erosion of the stapes.	1.1 x 0.7 cm (axial); 0.8 cm (cranial-caudal)	A
4	72	F	Centered at the jugular foramen, extending into the middle ear and eroding adjacent bone structures.	1.2 x 0.8 cm (axial); 1.5 cm (cranial-caudal)	C1
5	73	F	Occupying the hypotympanum, mesotympanum, and epitympanum. Involved ossicles and facial recess, minimal erosion of adjacent structures.	1.3 x 0.7 cm (axial); 1.5 cm (cranial-caudal)	B
6	76	F	Occupying the middle ear cavity, extending from the hypotympanum to the epitympanum and tympanic recess, closely abutting the ossicles without causing erosion.	1 x 0.5 cm (axial); 0.9 cm (cranial-caudal)	B
7	52	M	Intracranial extension. Invaded jugular foramen, mastoid, and temporal bone, with minor erosion into cochlear promontory and labyrinth.	2.7 x 2.2 cm (axial); 1.9 cm (cranial-caudal)	D1
8	75	F	Involving the right mastoid and middle ear, obstructing the external auditory canal.	2.7 x 1.2 cm (crosswise); 1.5 cm (cranial-caudal)	B
9	69	M	Occupying the middle ear cavity, extending from the hypotympanum to the epitympanum and tympanic recess, without causing erosion.	1.1 x 0.6 cm (axial); 1.1 cm (cranial-caudal)	B
10	64	F	Extending from the ossicles to the eustachian tube.	1.4 x 0.9 cm (axial); 1.4 cm (cranial-caudal)	A
11	62	F	Occupying most of the tympanic cavity without extension to the jugular foramen.	1.4 x 0.6 cm (axial); 1.2 cm (cranial-caudal)	B

**Figure 1:**
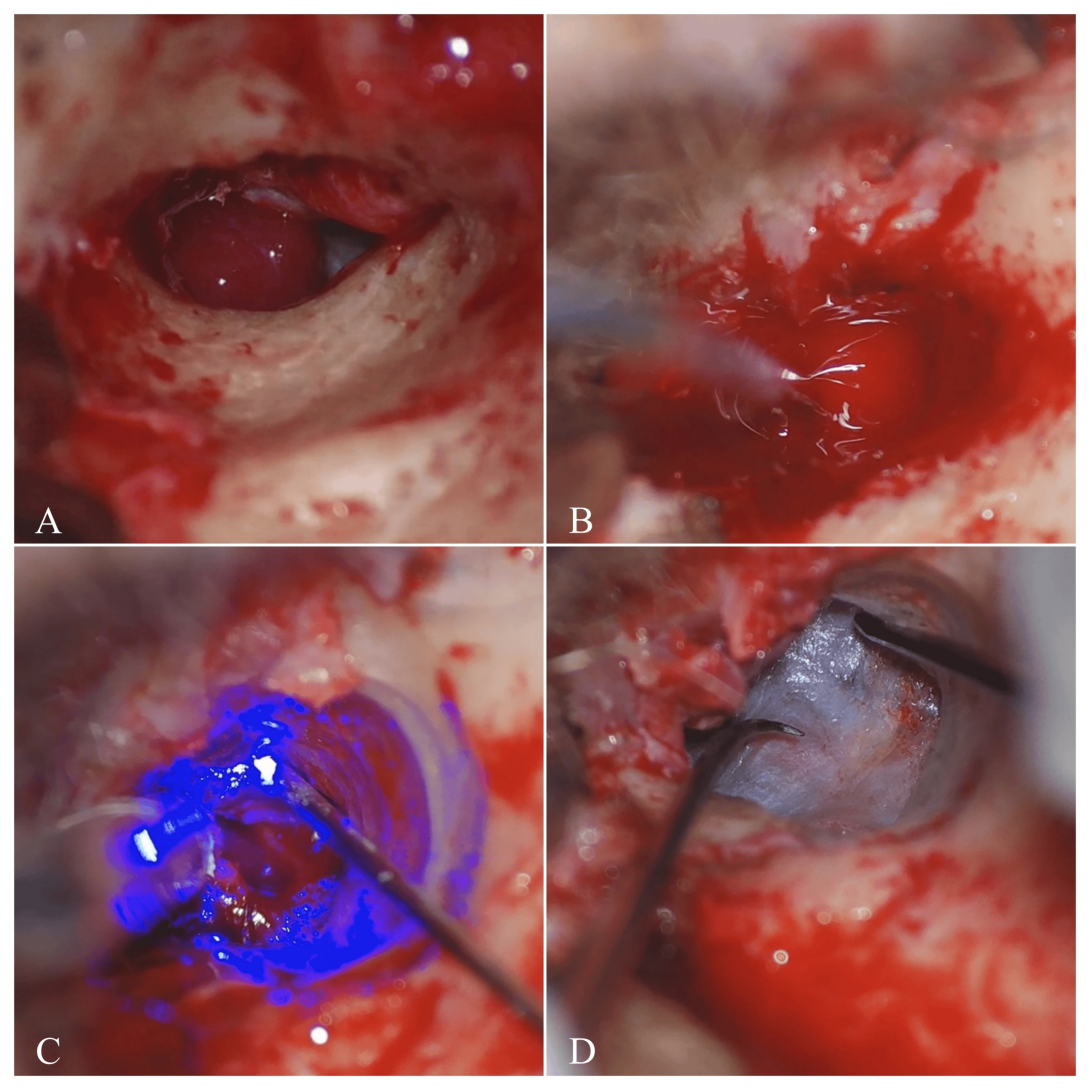
Surgical steps in case 6. (A) Exposure of the glomus tumor in the middle ear cavity after the elevation of the external auditory canal skin and the retraction of the tympanomeatal flap. (B, C) Potassium titanyl phosphate (KTP) laser (532 nm) is used to coagulate feeding vessels to minimize bleeding, while suction and irrigation ensure a clear surgical field. (D) Reposition of the tympanomeatal flap.

Surgical excision was the primary treatment for all patients. The approach varied based on tumor location and extent, with the endoaural-transcanal approach being the most common. Preoperative vascular embolization was not performed in any of the cases included in this series. A KTP laser was used for hemostasis in all surgeries (Figure 1).

In cases where the ossicles were eroded, they were removed. In several cases, ossicles such as the incus were sacrificed due to tumor infiltration, necessitating tympanoplasty for structural reconstruction, while the stapes was preserved. Extensive decompression or excision was required in one patient to address facial nerve involvement or significant bone erosion. Complete tumor removal was achieved in five patients (cases 3, 6, 9, 10, 11), with no recurrence observed over follow-up periods of up to two years. Residual tumors were documented in six patients (cases 1, 2, 4, 5, 7, 8), primarily due to tumor extension into critical structures. In all these cases, the residual tumor was located within the jugular foramen. To manage these cases, RS was selectively administered as an adjunctive treatment, contributing to either tumor stabilization or shrinkage. No postoperative complications or adverse effects related to radiotherapy were observed in any patient, including cranial nerve palsy, and no additional rehabilitation interventions were required. Follow-up HRCT and MRI results confirmed no recurrence in completely excised cases, with up to two years of stability. Postoperative worsening of conductive hearing loss was observed in cases involving ossicular chain erosion or removal (cases 1, 3, 5, 8, 10, 11), with an average deterioration in air conduction thresholds ranging from approximately 20 dB (cases 3, 5, 11) to 30 dB (cases 1, 8, 10). No patient exhibited worsening of sensorineural hearing loss following surgery, and no deterioration in sensorineural thresholds was recorded during follow-up. Histological analysis confirmed the diagnosis of paraganglioma in all cases, with a proliferation index (Ki-67) generally below 5%, indicating low tumor growth potential. However, a few tumors exhibited a higher Ki-67 index, including one with a 5% proliferation rate (Table 2).

**Table 2:** Treatment and outcomes. RS: radiosurgery.

Case	Surgery	RS	Histology: Proliferation index (Ki-67)	MRI follow-up
1	Partial excision plus type 4 tympanoplasty.	Yes	2-5%	2 years: Residual tumor near the cochlear promontory.
2	Partial excision with residual tumor left in the tympanic and facial recesses.	Yes	<3%	2 years: Residual tumor in the left jugular foramen. No clinical recurrence after RS.
3	Complete excision plus type 3 tympanoplasty.	No	<1%	2 years: No evidence of recurrence.
4	Extensive removal, spanning from the middle ear to the jugular foramen.	Yes	<2%	1 year: Reduction of tumor size with residual tumor in the left jugular foramen. 2 years: Stable residual tumor in the jugular foramen with no progression.
5	Partial excision. Left residual tumor tissue.	Yes	<3%	6 months: Residual tumor. 1 year: Significant tumor shrinkage after RS
6	Complete excision with preservation of ossicles plus type 1 tympanoplasty.	No	2–5%	1 year: No recurrence.
7	Extensive excision, decompression of the facial nerve.	Yes	<2%	2 years: Stable residual tumor in the jugular foramen with no progression.
8	Partial excision plus type 4 tympanoplasty (ossicles were corroded).	Yes	5%	1 year: No progression.
9	Complete excision.	No	<2%	1 year: Scar-like changes with no evidence of recurrence.
10	Complete excision requiring ossicular removal.	No	<2%	2 years: No recurrence, with stable findings consistent with surgical changes.
11	Complete tumor excision plus type 3 tympanoplasty.	No	<2%	1 year: No recurrence

## Discussion

Among the 11 patients included in our series, the majority were female, with a median age of 64.5 years, consistent with the known epidemiology of these tumors [1].

Several treatment strategies have been proposed in regards to temporal bone paragangliomas. Our study highlights the critical role of surgical excision as the cornerstone of primary treatment. Uddin et al. report that surgical excision is the standard of care for JTP [9]. Yildiz et al. propose surgery as a treatment option for patients with small tumors, due to a high control rate and fewer cranial nerve deficits compared to larger tumors [10]. This is confirmed by our results, as all tumors were less than 3 cm.

On the other hand, recent studies suggest considering RS as a primary treatment option [11-15]. Guss et al. conducted a meta-analysis on radiosurgical management and proposed RS as a viable alternative for glomus jugulare tumors [16]. In a study of 75 cases, Ibrahim et al. reported that Gamma Knife treatment led to tumor control in over 90% of patients, with minimal side effects and preservation of cranial nerve function [17]. Huy et al. advocate for RS as a first-line treatment, particularly for larger tumors or patients with no preoperative deficits [18]. Tse et al. further emphasize the role of stereotactic RS, showing that it stabilizes the disease and may lead to tumor size reduction [19]. Similarly, Shapiro et al. report that primary RS is safe and effective at controlling growth and clinical symptoms for patients with glomus jugulare [20]. A 2020 study on LINAC-treated patients demonstrated significant tumor reduction and excellent local control, offering an alternative to Gamma Knife for centers lacking that technology [21]. Similarly, Garg et al. reported tumor control rates exceeding 90% across different radiosurgical techniques, with no significant difference in efficacy among them [1].

Tumor location played a critical role in determining the surgical strategy. Based on our experience, glomus tympanicum tumors were more amenable to total excision, whereas glomus jugulare tumors often extended into critical neurovascular structures, requiring a more complex approach. Endoaural-transcanal approach, primarily utilizing cold steel microscopic middle ear surgery with KTP laser hemostasis, was the most commonly used in our series, as it is a viable and effective option, and its role in reducing surgical complications further supports its continued integration into surgical protocols. In certain cases, such as case 10, tumor infiltration necessitated the removal of ossicles, including the incus and stapes, requiring tympanoplasty for structural reconstruction. For patients with facial nerve involvement or significant bone erosion, extensive decompression was performed, as exemplified by case 7. Chaushu et al. report that both microscopic and endoscopic middle ear surgery yield favorable outcomes with low complication rates in temporal bone paraganglioma resection [22]. Additionally, Salem et al. suggest that coblation serves as an effective and safe technique for excising glomus tympanicum tumors [23]. Given the vascularity of JTP, other hemostatic methods, such as endoscopic bipolar cautery and preoperative embolization, have also been recommended [9,22,24].

The findings from this study highlight the direct correlation between Fisch classification and treatment outcomes. Class A tumors (cases 3, 10), which were confined to the tympanic cavity, were fully resected, requiring no additional treatment, and exhibited no recurrence on follow-up. In contrast, class B tumors (cases 1, 2, 5, 6, 8, 9, 11), which involved the middle ear without jugular foramen invasion, showed mixed outcomes, with smaller lesions being completely excised, while larger or more complex tumors required adjuvant therapy. Among class B tumors, case 5 demonstrated significant tumor shrinkage after RS, whereas cases 1, 2, and 8 had stable residual disease without progression. More advanced cases, such as class C1 (case 4) and class D1 (case 7), which extended into the jugular foramen and intracranial structures, respectively, required extensive surgical excision but could not be fully resected, leaving residual tumors that were effectively stabilized with RS, preventing further progression at two-year follow-up. While surgical excision remains the gold standard, RS is essential for managing residual disease, particularly in cases where complete resection is unfeasible due to cranial nerve involvement or skull base extension. RS offers a favorable safety profile, with low risks of nerve damage, hemorrhage, or complications associated with skull base surgery [25]. Specifically, our findings confirm prior reports, such as those by Al-Mefty et al., who noted that glomus tympanicum tumors are often resectable with minimally invasive techniques, whereas glomus jugulare tumors require extensive surgery, often combined with RT, especially in cases with cranial nerve or brainstem compression [26]. Similarly, Yildiz et al. reported that while patients with Fisch C and D temporal bone paragangliomas can undergo surgical treatment, subtotal resection is often the only viable option, leading the authors to propose a multimodal approach combining surgery and RS for larger tumors [10]. Additionally, Spector et al. demonstrated long-term tumor control in patients treated with surgery followed by RT, leading to either tumor stabilization or reduction [7].

Histological analysis confirmed that most tumors had a low Ki-67 proliferation index (<3%), consistent with their slow-growing nature. However, in cases 1, 6, and 8, a Ki-67 proliferation rate of 2-5% was not associated with tumor progression, further emphasizing that proliferation markers alone do not guide treatment decisions. Ki-67 is a nuclear protein and a widely used proliferation marker that plays a crucial role in assessing tumor growth activity, including in paragangliomas [27-28]. While these tumors are generally slow-growing, they can exhibit varying degrees of aggressiveness. Higher Ki-67 labeling indices have been linked to increased tumor cell proliferation and, in some cases, a greater likelihood of recurrence or metastatic potential, particularly in temporal bone paragangliomas [29]. However, the clinical significance of Ki-67 in paragangliomas remains an area of ongoing research. Some studies suggest that Ki-67 levels above 3-5% may indicate a more aggressive phenotype, but no universally accepted cutoff value reliably differentiates benign from malignant behavior [30]. Our findings support that while Ki-67 may offer prognostic insights, it should always be interpreted within a broader clinical and pathological context.

**Figure 2:**
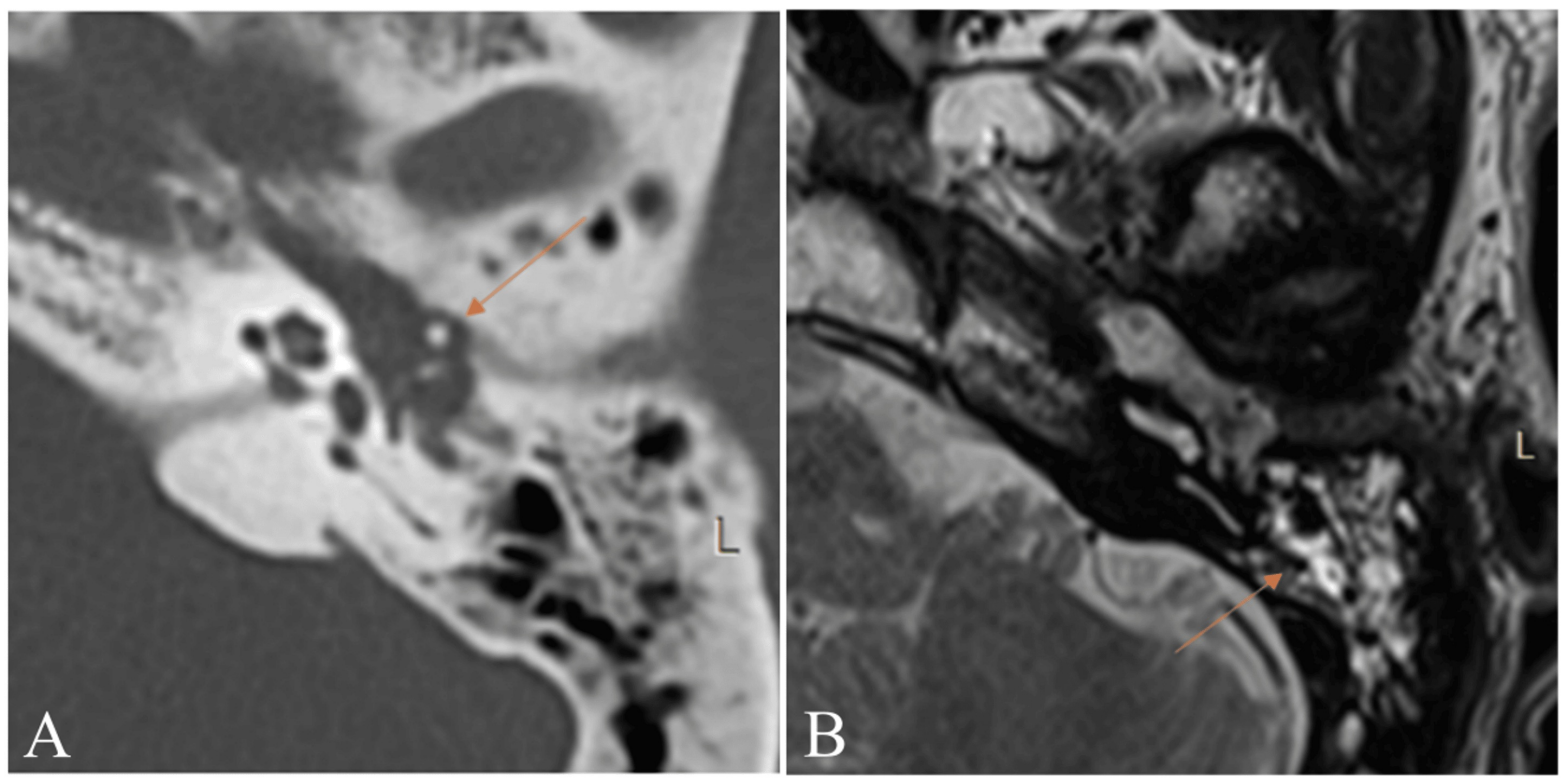
Imaging of case 1. (A) High-resolution axial CT of the internal auditory canal (IAC) without contrast showing a tumor surrounding the left ossicular chain. (B) T2-weighted MRI showing a tumor in the left middle ear cavity.

**Figure 3:**
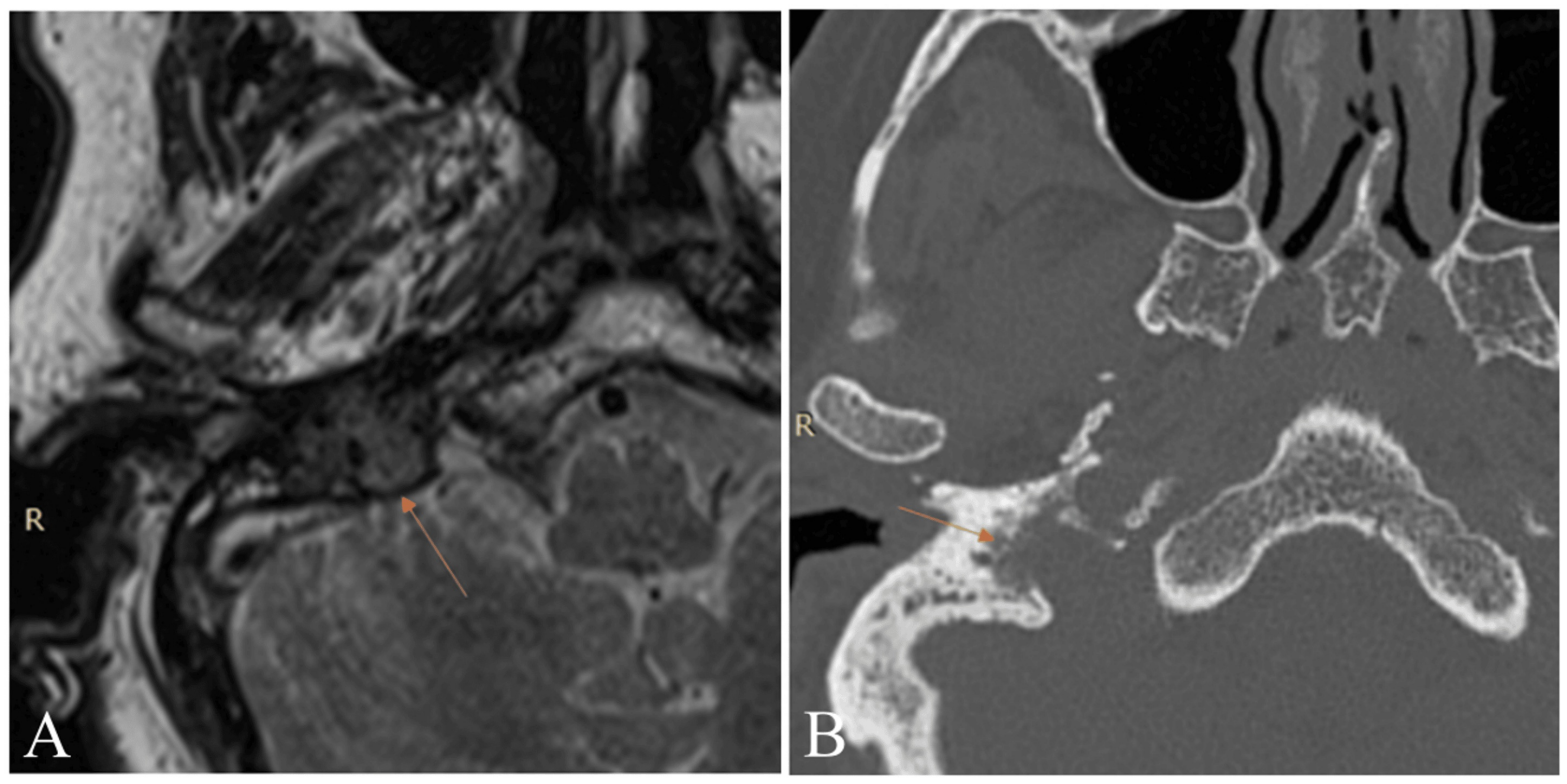
Imaging of case 8. (A) T2-weighted MRI (arrow) showing a vascular tumor in the right jugular foramen with flow voids (salt and pepper sign). (B) High resolution axial CT of the internal auditory canal (IAC) without contrast showing the tumor eroding the jugular foramen walls.

In our series, all patients were diagnosed, staged, and followed up with HRCT and gadolinium-enhanced MRI, which remain a critical imaging modality in JTP management [2]. HRCT is particularly valuable for assessing bony erosion, jugular foramen widening, and skull base involvement, which are characteristic of these highly vascular tumors. It provides high-resolution images of the osseous structures, helping to distinguish JTP from other middle ear masses. MRI, on the other hand, is the modality of choice for evaluating the soft tissue extent, vascularity, and neurovascular involvement [31]. T2-weighted MRI typically reveals the classic “salt-and-pepper” appearance, representing hyperintense tumor stroma ("salt") and hypointense flow voids from high-velocity blood flow ("pepper") (Figures 2, 3).

While our study includes a follow-up period of up to two years, prior research has reported that a combined approach can achieve tumor stabilization, size reduction, and prevention of further growth in up to 95% of cases, with long-term follow-up studies extending beyond 10 years [24]. Long-term studies also confirm that patients maintain a good quality of life post treatment, with minimal severe side effects.

Limitations

Despite these valuable insights, our study has limitations. The small sample size restricts generalizability, and the retrospective nature introduces potential biases in data collection. Additionally, the follow-up period, though extending up to two years, may not be sufficient to fully assess long-term recurrence patterns. Larger, multicenter studies are needed to validate these observations.

## Conclusions

This study highlights the importance of an individualized treatment approach for jugulotympanic paragangliomas. Surgical excision remains the gold standard for glomus tympanicum and smaller, well-localized tumors, offering complete removal with minimal morbidity. However, for glomus jugulare tumors and patients with unresectable lesions or high surgical risks, a combined approach using surgery and RS is well established. RS serves as a non-invasive alternative, effectively leading to tumor stabilization or shrinkage while minimizing the risks of nerve damage and surgical complications. HRCT and MRI continue to play a crucial role in both initial diagnosis and long-term follow-up, enabling precise assessment of tumor extent and treatment response. While the Ki-67 proliferation index offers insights into tumor growth potential, it does not solely determine recurrence risk, emphasizing the need for a comprehensive clinical and imaging-based approach to treatment planning.
